# Rituximab utilization for approved and off‐label nononcology indications and patients’ experiences with the Patient Alert Card

**DOI:** 10.1002/prp2.555

**Published:** 2020-01-03

**Authors:** Khaled Sarsour, Senam Beckley‐Kartey, Simone Melega, Adefowope Odueyungbo, Petra Kirchner, Natasha Khalife, Joanne Bangs

**Affiliations:** ^1^ Genentech, Inc South San Francisco CA USA; ^2^ F. Hoffmann‐La Roche Ltd Basel Switzerland; ^3^ Roche Products Ltd Mississauga ON Canada; ^4^ IQVIA Reading UK; ^5^ Joanne Bangs Ltd Letchworth Garden City Hertfordshire UK; ^6^ Present address: Fulcrum Therapeutics Cambridge MA USA; ^7^ Present address: IQVIA Dubai United Arab Emirates

**Keywords:** anti‐CD20, infection, off‐label, Patient Alert Card, progressive multifocal leukoencephalopathy, rituximab

## Abstract

This study used retrospective chart review and survey data to evaluate: (1) off‐label use of rituximab (MabThera^®^/Rituxan^®^) in autoimmune conditions and (2) patients’ receipt and knowledge of the Patient Alert Card (PAC), a risk minimization measure for progressive multifocal leukoencephalopathy (PML) and serious infections. Anonymized patient data were collected from infusion centers in Europe from December 2015 to July 2017. Adults receiving rituximab in the same centers were provided a self‐administered survey. Outcomes included patterns of off‐label rituximab use for nononcology indications, and evaluation of patients’ receipt and knowledge of the PAC and its impact. Of 1012 patients in the retrospective chart review, 70.2% received rituximab for rheumatoid arthritis or granulomatosis with polyangiitis/microscopic polyangiitis, and 29.8% received rituximab off label. Among 524 survey participants, 32.8% reported receiving the PAC, 59.3% reported not receiving the PAC and 7.9% did not know whether they received the PAC. A total of 72.4% of patients reported that they were unaware that some patients receiving rituximab experience PML. A higher proportion of PAC recipients identified PML as a potential risk of rituximab than nonrecipients (37.8% vs 19.9%); 58.3% of PAC recipients had poor awareness of PML. Most PAC recipients (90.0%) and nonrecipients (85.5%) correctly answered that they should seek medical attention for infection symptoms. In conclusion, approximately 30% of patients received off‐label rituximab. Most patients reported not receiving the PAC or having knowledge of PML but demonstrated understanding of the recommended action in the event of infection symptoms, regardless of PAC receipt.

**Abbreviations**
GPA: granulomatosis with polyangiitis
HCPs: healthcare providers
MPA: microscopic polyangiitis
PAC: Patient Alert Card
PML: progressive multifocal leukoencephalopathy
RA: rheumatoid arthritis

## 1 INTRODUCTION

Rituximab (MabThera^®^/Rituxan^®^), a chimeric monoclonal antibody that targets and depletes CD20‐positive B cells, has a safety profile that is well characterized and established in the approved oncology indications (non‐Hodgkin's lymphoma, chronic lymphocytic leukemia) and autoimmune indications (rheumatoid arthritis [RA], granulomatosis with polyangiitis [GPA] and microscopic polyangiitis [MPA], and pemphigus vulgaris [newly approved]).^1^, ^2^ Due to its mechanism of action, rituximab is also used off label by healthcare providers (HCPs) to treat other autoimmune conditions,^3^, ^4^, ^5^, ^6^, ^7^ often in patients who are refractory to approved treatments.

Because B‐cell depletion may lead to a suppressed immune system, patients receiving rituximab may have an increased risk of infections, including serious infections and progressive multifocal leukoencephalopathy (PML). These are two of the well‐known identified risks of rituximab in all approved indications,^2^ although an association between the occurrence of PML and the extent of rituximab exposure, with any mechanistic association between B‐cell depletion and John Cunningham virus (JCV) reactivation, remains unclear. PML is a very rare, often fatal event among rituximab‐treated patients with RA or GPA/MPA, and its occurrence has remained stable over time.^8^ PML rates have been reported as 2.56 per 100,000 patients with RA who have received rituximab and <1 per 10 000 patients with GPA/MPA.^8^ In all reported cases, the patients had ≥1 risk factor for PML independent of rituximab treatment including prior and concomitant therapies, a history of malignancy, prior or concomitant SLE, and other immune disorders (leukopenia, lymphopenia).^8^

Following reports of PML in patients treated with rituximab, an additional risk minimization measure was requested by the European Medicines Agency (EMA). A Patient Alert Card (PAC)^9^ focusing on the potential increased risks of PML and other infections was implemented in 2009 and extended to all nononcology indications following the approval of rituximab for the treatment of GPA/MPA in 2013. The PAC is supplied to the HCPs for provision to patients by two routes: directly to the HCPs via the local company affiliates and attached to the rituximab package leaflet within the drug carton.

The purpose of the PAC is to inform the patient of the need for vigilance with respect to PML and other infections generally. Furthermore, the objective of the PAC is to ensure that patients seek medical attention early and that HCPs are aware of the need for timely and appropriate measures to diagnose PML. The rationale is that, with a timely diagnosis of PML or infection, treatment with rituximab could be discontinued and reductions or discontinuation of concomitant immunosuppressive therapy considered. Reconstitution of the immune system in immunocompromised patients with PML has resulted in stabilization or improved outcome.^10^ Whether early detection of PML and suspension of rituximab therapy may lead to similar stabilization or improved outcome is unknown.^2^, ^11^

The aims of this study were: (1) to quantify and characterize off‐label use of rituximab by evaluating the medical records of patients treated with rituximab for nononcology conditions, and (2) to use survey data to assess the extent to which patients receive and read the PAC, their knowledge of the PAC content, and whether distribution of the PAC might influence patient actions.

## 2 MATERIALS AND METHODS

### 2.1 Medical record data collection to determine off‐label use

A total of 47 infusion centers (defined as centers where infusions of rituximab [MabThera] may have taken place) in France, Germany, Italy, Spain, and the United Kingdom were recruited to participate in the study. At each participating infusion center, anonymized patient data were collected from 17 December 17 2015, to 7 July 2017. Data included age, sex, condition for which rituximab were prescribed, reason for rituximab prescription, date of first diagnosis of the condition, severity of RA using Disease Activity Score based on 28 joints (DAS28), presence of extra‐articular involvement, C‐reactive protein level, rituximab dosage for the most recent infusion, number of rituximab infusions in the past 2 years, and other current and previous antiinflammatory medications.

Medical records data were stratified by indication (RA, GPA/MPA, systemic lupus erythematosus [SLE], and other), prior use of tumor necrosis factor inhibitors (patients with RA only), country, number of infusions received, PAC receipt (survey patients only), sex, age groups (18‐45, 46‐65, and >66 years), duration of rituximab treatment, level of education (survey patients only), most recent infection (survey patients only), and selected patient and disease characteristics for all patients receiving rituximab for a nononcology condition.

**Figure 1:**
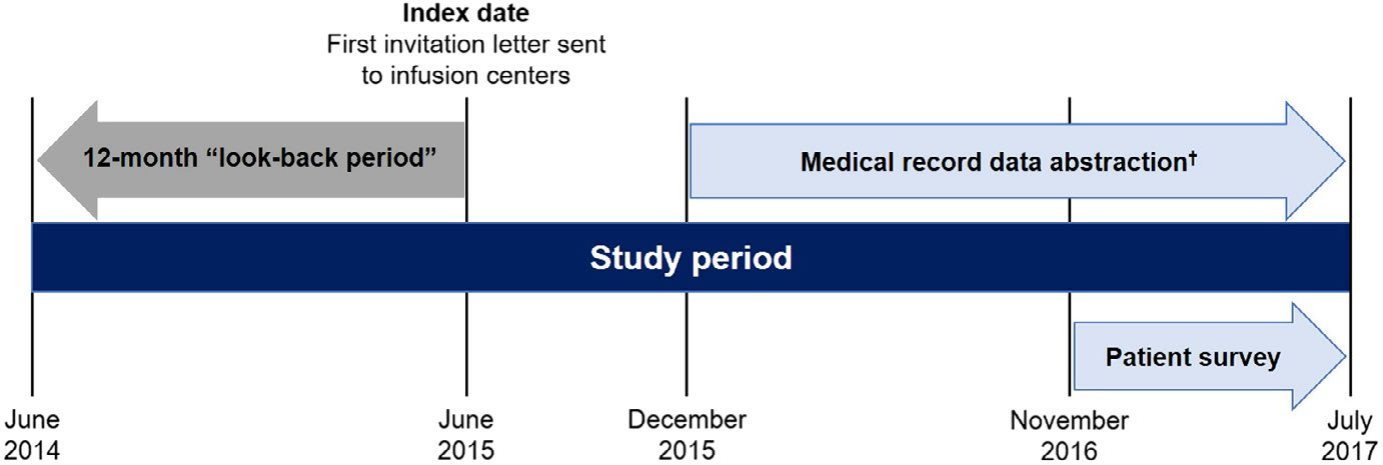
Study Design. ^†^ Data abstraction of medical records spanning June 2014 to June 2015 (“look‐back period”)

The study index date, 19 June 2015, corresponded to the date the first invitation letter was sent to infusion centers. Medical records data from a period of 12 months prior to index date were retrospectively reviewed and abstracted (Figure 1). This “look‐back period” from June 2014 to June 2015 was used to ensure that data collected were fully reflective of the real‐world administration of rituximab and not influenced by study awareness. The observation period corresponded to the interval between the first and last infusion dates of rituximab, if treatment was discontinued before the index date. If the patient was still receiving rituximab at the index date, then the observation period was the interval between the first infusion date and the index date.

This study was conducted in accordance with all applicable ethical and regulatory requirements, including the Declaration of Helsinki. Approval from each relevant ethics committee was obtained prior to the study start in each country and documented in a letter to the center specifying the date on which the ethics committee granted approval. All patients provided informed consent for participation in the medical records data collection and/or survey.

### 2.2 Patient Alert Card survey

Patients aged ≥ 18 years receiving rituximab for a nononcology indication were recruited from November 2016 to July 2017 from the same infusion centers participating in the medical records data collection and provided with a self‐administered survey (Figure 1). Patients were excluded if they had participated in a clinical trial in which rituximab was one of the treatments evaluated. Patients were permitted to complete the survey only once. The survey consisted of prospective collection of information on patient characteristics, including questions about patient knowledge of the risks of PML and other infections, patient receipt and review of the PAC, and any actions the patient would take or had taken as a result of receiving the PAC.

### 2.3 Safety

Individual adverse event (AE) information captured during the survey was reported to Roche as the Marketing Authorization Holder (MAH).

### 2.4 Statistical analysis

Analyses of both rituximab off‐label use (for indications other than RA or GPA/MPA; the pemphigus vulgaris indication was not approved at the time of this study) and the evaluation of patient receipt and knowledge of the PAC were descriptive in nature and included summary statistics and the frequency distribution of item responses. No statistical testing was performed; however, 95% CIs of the proportions of patients receiving rituximab for approved and off‐label uses were calculated to assess precision of the prevalence estimates. Missing data were not imputed, and the data were analyzed and presented as recorded.

## 3 RESULTS

### 3.1 Medical records data for off‐label use

A total of 1012 patients from 47 centers were included in the retrospective data collection. Patients were predominantly female (75.5%) and mainly fell within the age categories of 46 to 55 years (21.6%), 56 to 65 years (26.1%) and 66 to 75 years (26.5%). Overall, mean (SD) and median interquartile range (IQR) time from diagnosis of the primary condition until data abstraction were 11.0 (8.17) years and 8.5 (4.7‐15.5) years, respectively. Mean (SD) and median (IQR) time since first rituximab infusion were estimated at 43.7 (28.8) months and 32.9 (21.3‐61.8) months, respectively.

Among the 1012 patients, 710 (70.2%) received rituximab (MabThera) for an approved nononcology indication: 618 patients (61.1%) with RA and 92 patients (9.1%) with GPA/MPA. A total of 302 patients (29.8%) received rituximab for an off‐label nononcology indication; the most common conditions for prescribing rituximab off label were SLE (58 patients [5.7%]) and Sjögren's syndrome (49 patients [4.8%]) (Table 1).

**Table 1:** Primary condition for prescribing rituximab^a^

Primary condition, n (%)	France n = 204	Germany n = 212	Italy n = 198	Spain n = 198	United Kingdom n = 200	Total N = 1012
Approved indication						
RA	121 (59.3)	115 (54.2)	93 (47.0)	124 (62.6)	165 (82.5)	618 (61.1)
GPA/MPA	22 (10.8)	44 (20.8)	19 (9.6)	5 (2.5)	2 (1.0)	92 (9.1)
Off‐label indication						
Other^b^	37 (18.1)	21 (9.9)	20 (10.1)	13 (6.6)	13 (6.5)	104 (10.3)
SLE	6 (2.9)	9 (4.2)	10 (5.1)	18 (9.1)	15 (7.5)	58 (5.7)
Sjögren's syndrome	5 (2.5)	2 (0.9)	31 (15.7)	10 (5.1)	1 (0.5)	49 (4.8)
Systemic vasculitis	3 (1.5)	3 (1.4)	7 (3.5)	4 (2.0)	1 (0.5)	18 (1.8)
Eosinophilic granulomatosis with polyangiitis	1 (0.5)	5 (2.4)	7 (3.5)	0	1 (0.5)	14 (1.4)
Polydermatomyositis	2 (1.0)	3 (1.4)	1 (0.5)	8 (4.0)	0	14 (1.4)
Mixed connective tissue disease	1 (0.5)	1 (0.5)	1 (0.5)	7 (3.5)	1 (0.5)	11 (1.1)
Nephrotic syndrome	2 (1.0)	1 (0.5)	7 (3.5)	0	0	10 (1.0)

Abbreviations: GPA, granulomatous with polyangiitis; MPA, microscopic polyangiitis; RA, rheumatoid arthritis; SLE, systemic lupus erythematosus.

a Conditions summarized in the table were recorded in ≥1% of patients.

b Other off‐label indications included: ankylosing spondylitis, psoriatic arthritis, juvenile idiopathic arthritis, systemic vasculitis, inflammatory myopathies, Behçet disease, nephrotic syndrome, glomerulonephritis, multiple sclerosis/neuromyelitis optica, polydermatomyositis, mixed connective tissue disease, eosinophilic granulomatosis with polyangiitis, and other (undefined).

Demographics were similar between groups of patients who received rituximab off label and for approved indications (data not shown). Failure of previous treatment was the most common reason for rituximab prescription in patients treated off label and in those treated for approved indications, recorded in 231/302 patients (76.5%) and 588/710 patients (82.8%), respectively (Table 2). A greater proportion of patients receiving rituximab for an off‐label indication received rituximab for <5 years than did those receiving rituximab for approved indications (256/292 [87.7%] vs 464/675 [67.6%], respectively). The median (IQR) number of individual rituximab infusions in the 2 years prior to the index date was 4.0 (2.0‐5.0) infusions in both patients treated off label and those treated for approved indications.

**Table 2:** Characteristics of off‐label and approved rituximab use

	Off‐Label Use n = 302	Approved Use n = 710	Total N = 1012
Reason for rituximab prescription, n (%) [95% CI]			
Failure of previous treatment	231 (76.5) [71.7, 81.3]	588 (82.8) [80.0, 85.6]	819 (80.9) [78.5, 83.4]
AEs under previous treatment	10 (3.3) [1.3, 5.3]	46 (6.5) [4.7, 8.3]	56 (5.5) [4.1, 6.9]
Compassionate use	23 (7.6) [4.6, 10.6]	22 (3.1) [1.8, 4.4]	45 (4.4) [3.2, 5.7]
Data not available	14 (4.6) [2.3, 7.0]	14 (2.0) [0.9, 3.0]	28 (2.8) [1.8, 3.8]
Other	24 (7.9) [4.9, 11.0]	40 (5.6) [3.9, 7.3]	64 (6.3) [4.8, 7.8]
Duration since rituximab initiation, n (%) [95% CI]			
n	292	675	967
<5 y	256 (87.7) [83.9, 91.4]	456 (67.6) [64.0, 71.1]	712 (73.6) [70.9, 76.4]
≥5 y	36 (12.3) [8.6, 16.1]	219 (32.4) [28.9, 36.0]	255 (26.4) [23.6, 29.1]
Total number of individual rituximab infusions in the past 2 y			
n	302	707	1009
Mean (SD)	3.8 (2.3)	3.9 (2.0)	3.9 (2.0)
Median (IQR)	4.0 (2.0‐5.0)	4.0 (2.0‐5.0)	4.0 (2.0‐5.0)

Abbreviations: AE, adverse event; IQR, interquartile range.

### 3.2 Patient Alert Card survey

**Figure 2:**
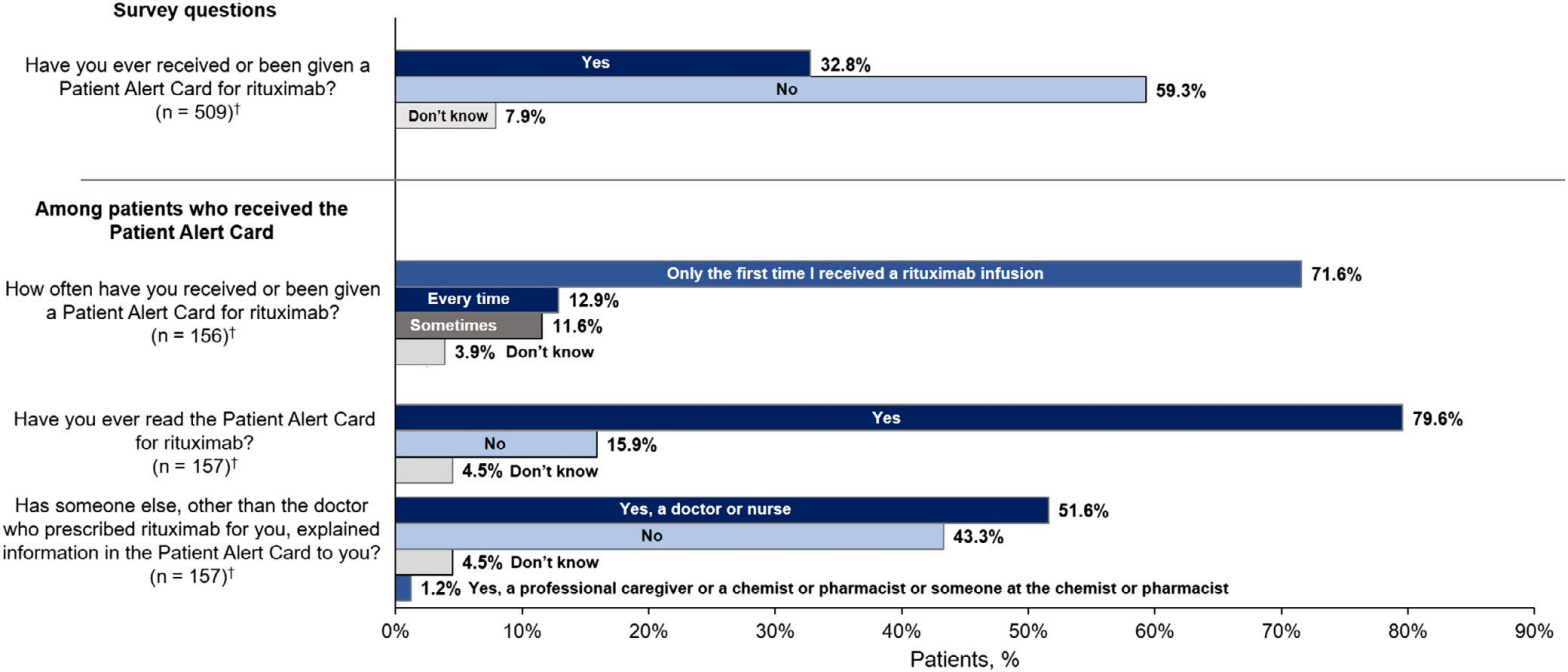
Patients’ Receipt and Review of the PAC. PAC, Patient Alert Card. ^†^ Number of patients who answered the question

A total of 524 patients participated in the patient survey; the numbers of evaluable patients for the individual survey questions varied due to the fact that not all patients answered every survey question. Most patients were female (382/519 [73.6%]) and fell within age categories of 46‐55 years (113/519 [21.8%]), 56‐65 years (147/519 [28.3%]), and 66‐75 years (124/519 [23.9%]). A total of 167/509 patients (32.8%) reported they received the PAC, 302 (59.3%) reported they did not receive the PAC, and 40 (7.9%) did not know whether they had received the PAC (Figure 2). Among patients who reported receiving the PAC and answered the following questions, 111/155 (71.6%) responded that they received the PAC only the first time they received a rituximab infusion, 81/157 (51.6%) reported that they received an explanation of the PAC content from a doctor or nurse other than the doctor who prescribed rituximab, and 125/157 (79.6%) reported they had read the PAC (Figure 2).

**Figure 3:**
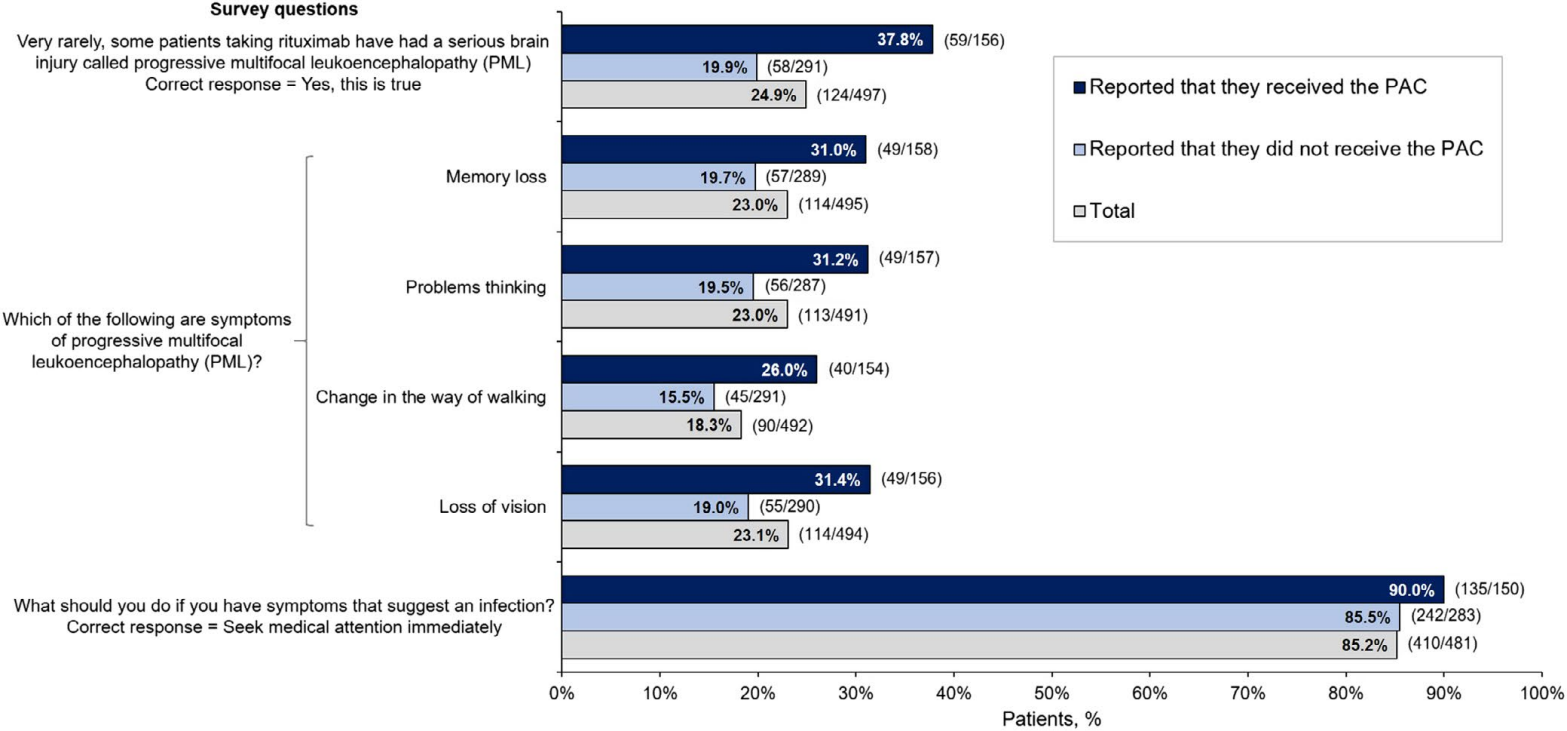
Proportion of Patients With Correct Responses to Key Knowledge Questions by PAC Receipt. PAC, Patient Alert Card

Only 124/497 patients (24.9%) reported that they were aware that, very rarely, some patients being treated with rituximab experience PML (Figure 3). A greater proportion of patients who reported that they received the PAC correctly identified PML as a potential side effect of rituximab than patients who reported that they had not received the PAC (59/156 [37.8%] vs 58/291 [19.9%], respectively). A total of 91/156 patients (58.3%) answered “I don't know” in response to the question asking if, very rarely, some patients receiving rituximab experience PML.

The proportions of patients who correctly identified four possible symptoms of PML were as follows: memory loss, 114/495 (23.0%); problems thinking, 113/491 (23.0%); change in the way of walking, 90/492 (18.3%), and loss of vision, 114/494 (23.1%) (Figure 3). Each PML symptom was correctly identified by a greater proportion of PAC recipients than nonrecipients, as shown in Figure 3 **.** Regarding the action to be taken if experiencing symptoms suggestive of infection, the majority of respondents (410/481 [85.2%]) answered correctly that they should seek medical attention immediately. The correct answer was selected by 135/150 PAC recipients (90.0%) and 242/283 PAC nonrecipients (85.5%).

In response to the question about the action previously taken when the patient experienced their most recent infection, 49/70 patients (70.0%) answered, “When I noticed symptoms, I talked to my doctor”; the next most common response was, “I told the doctor who treated me for the infection that I was taking rituximab” (28/70 [40.0%]). A greater proportion of patients who reported that they received the PAC responded “When I noticed symptoms, I talked to my doctor” than patients who reported that they had not received the PAC (22/28 [78.6%] vs 25/39 [64.1%], respectively).

### 3.3 Safety

A total of 125 AEs, of which 14 were serious, were reported by 87/547 patients (15.9%) who took the survey. No cases of PML were reported. The AEs reported were consistent with the known rituximab safety profile in these indications; based on these data, no new safety signals were identified.^2^

## 4 DISCUSSION

In this study, rituximab was used off label in approximately 30% of patients. Previous studies have demonstrated that rituximab is used off label to treat a range of autoimmune diseases.^3^, ^12^, ^13^ Furthermore, a systematic review conducted by the European Commission showed that the off‐label use of rituximab was particularly high in patients with autoimmune disease.^14^ The off‐label use of rituximab in this study was consistent with the known pattern and extent of off‐label use of rituximab for autoimmune conditions, with no new safety signals detected.^3^

Among the patients surveyed, most reported that they did not receive the PAC. This study did not investigate the reasons why patients did not receive the PAC, but possible barriers to PAC distribution include the PAC not being passed from the pharmacy to the patients or a HCP decision not to provide the PAC. Although a higher proportion of PAC recipients identified PML as a potential risk of rituximab than nonrecipients, overall, patients had poor knowledge of PML, showing that the PAC may only contribute to patient knowledge to a limited extent. These results were consistent with those of a previous study evaluating the effectiveness of a PAC in educating patients receiving natalizumab on the risk of PML, which showed that only 16/37 patients (43.2%) who received the PAC answered all PML basic knowledge questions correctly.^15^ Reaching more patients with the PAC information and improving knowledge retention are important goals for HCPs, patients, regulators, and MAHs.

Regardless of PAC receipt, patients demonstrated an understanding of the recommended action to take in the event of infection symptoms. Patients’ awareness of PML and the steps to take in case of infection symptoms could have resulted from receipt of information from other sources. For example, educational material to further inform patients with RA and GPA/MPA who receive rituximab on the risks of PML and other infections is in place as an additional risk minimization measure. Understanding the most effective means of educating patients about the risks of therapies and ensuring patient engagement in education is a key area of discussion.

### 4.1 Limitations

Findings of this study largely pertain to the infusion centers involved in routine rheumatological practice that predominantly (≈80%) participated in this study; therefore, certain off‐label conditions not primarily treated in routine rheumatology practice (eg, some dermatologic or ophthalmologic conditions) were unlikely to be captured in this study.

As is the case with all voluntary surveys, invited patients self‐selected into the survey component of the study, and thus selection bias may have led to an underestimate or overestimate of the level of patient understanding. Overall, there was a very good response rate to individual questions (approximately ≥90% response rate per question, in general); however, a small number of questions were answered by fewer patients. For example, the question asking patients to indicate the most recent infection experienced was answered by only approximately 60% of patients; the low response rate may be due to a lack of patient understanding of the type of infection they experienced, or due to some patients not remembering the type of infection they experienced.

Furthermore, patients’ answers to the survey may have been influenced by when they received the PAC and their ability to recall the information contained in the PAC. For example, 71.6% of patients reported receiving the PAC only the first time they received a rituximab infusion, and only 12.9% of patients reported that they received the PAC every time they received a rituximab infusion. Therefore, if patients had not received the PAC when they arrived at the clinic (the survey was administered after arrival but before the infusion) but had received it at their last infusion (weeks to months previously), they may not recall if they had received the PAC or the information it contained. Finally, all analyses were descriptive, with no correlations noted or statistical significance tests performed; therefore, it is not possible to confirm an association between PAC receipt and a greater understanding of the risks of PML and other infections.

Results of this study demonstrated that in nononcology conditions, rituximab was used predominantly in indications approved at the time of the study: RA and GPA/MPA. Off‐label use was in line with the known pattern and extent of off‐label use of rituximab for autoimmune conditions, with no new safety signals detected.

The patient survey showed that most patients reported that they did not receive the PAC, and the results indicate slightly better knowledge scores among patients who reported that they received the PAC compared to those who reported that they did not receive the PAC. Although PAC recipients did not show greater knowledge of the risk or symptoms of PML, overall, patients demonstrated an understanding of the recommended action to take in the event of infection symptoms, regardless of PAC receipt.

## 5 ETHICS STATEMENT

This study was conducted in accordance with all applicable ethical and regulatory requirements, including the Declaration of Helsinki. Approval from each relevant ethics committee was obtained prior to the study start in each country and documented in a letter to the center specifying the date on which the ethics committee granted approval. All patients provided informed consent for participation in the medical records data collection and/or survey.

## DISCLOSURES

The study sponsor, F. Hoffmann‐La Roche Ltd, was involved in the study design, collection, analysis, and interpretation of the data, the writing of the manuscript, and the decision to submit the manuscript for publication. K. Sarsour is an employee of Genentech, Inc. S. Beckley‐Kartey, S. Melega, and P. Kirchner are employees of F. Hoffmann‐LaRoche Ltd. A. Odueyungbo was an employee of Roche Products, Ltd, at the time of this study. N. Khalife is an employee of IQVIA. J. Bangs was an independent regulatory consultant to F. Hoffmann‐LaRoche Ltd at the time of the study.

## AUTHORS' CONTRIBUTIONS

K. Sarsour, S. Beckley‐Kartey, S. Melega, A. Odueyungbo, and J. Bangs were involved in drafting, reviewing, and revising the manuscript. P. Kirchner and N. Khalife were involved in reviewing and revising the manuscript critically. K. Sarsour, S. Beckley‐Kartey, S. Melega, A. Odueyungbo, and P. Kirchner contributed to the conception and study design, acquisition, analysis, and interpretation of data. N. Khalife and J. Bangs contributed to the study design, analysis, and interpretation of data. All authors read and approved the final manuscript.

## Data Availability

Access to individual patient level data from the datasets used and/or analyzed during the current study may be requested by qualified researchers through the clinical study data request platform (http://www.clinicalstudydatarequest.com). Further details on Roche's criteria for eligible studies are available here: (https://clinicalstudydatarequest.com/Study-Sponsors/Study-Sponsors-Roche.aspx). For further details on Roche's Global Policy on the Sharing of Clinical Information and how to request access to related clinical study documents, see here: (https://www.roche.com/research_and_development/who_we_are_how_we_work/clinical_trials/our_commitment_to_data_sharing.htm).
